# Melanoma Incidence, Mortality, and Dermatologist Availability Across Selected Urban and Rural Counties in Southwest Missouri

**DOI:** 10.7759/cureus.105007

**Published:** 2026-03-10

**Authors:** George Ladas, Derek S Towery

**Affiliations:** 1 Medicine, Kansas City University of Medicine and Biosciences, Kansas City, USA; 2 Dermatology, U.S. Dermatology Partners, Joplin, USA

**Keywords:** access to health care, dermatologist availability, melanoma, missouri, rural health disparities, skin cancer epidemiology

## Abstract

Melanoma is the most lethal of the common skin cancers, and its incidence continues to rise in the United States. Access to dermatologic specialty care remains below recommended benchmarks, particularly in rural and nonmetropolitan regions. This descriptive, population-based analysis examined melanoma incidence and dermatologist availability across five selected counties in southwest Missouri, with limited county-level mortality reporting. Age-adjusted melanoma incidence data were obtained from the State Cancer Profiles database (National Cancer Institute and Centers for Disease Control and Prevention) and standardized to the 2000 U.S. population. Dermatologist availability was calculated using National Provider Identifier data and reported as dermatologists per 100,000 population. Nonmetropolitan counties demonstrated comparable or higher melanoma incidence rates alongside substantially lower dermatologist density; however, findings are descriptive and do not establish causal relationships. Melanoma mortality could not be meaningfully compared across counties, as data were suppressed in multiple counties due to low case counts. Greene County, designated as the urban reference county, had the highest dermatologist density and was the only county with non-suppressed mortality data. These findings highlight geographic variation in melanoma incidence and provider distribution while underscoring the challenges of interpreting melanoma burden in sparsely populated regions.

## Introduction

Melanoma is a malignant transformation of melanocytes and represents the most lethal of the common skin cancers, accounting for the majority of skin cancer-related deaths, with incidence rates continuing to increase [1,2]. Early detection and timely treatment of melanoma are critical, as prognosis is strongly associated with tumor stage at diagnosis [3]. Routine skin examinations and prompt evaluation, therefore, play a central role in improving melanoma outcomes.

Access to dermatologic specialty care varies substantially based on socioeconomic status, rurality, and the geographic distribution of dermatology providers [4]. Lower socioeconomic status and residence in a rural location create barriers to medical care, including longer wait times, transportation difficulties, and insurance-related limitations [4]. Moreover, rural populations are more likely to work in outdoor occupations, leading to increased lifetime sun exposure and potentially higher melanoma risk [3]. This elevated risk, coupled with rising melanoma incidence rates, underscores a growing demand for dermatologic care; however, workforce distribution remains uneven. Although the recommended dermatologist density is approximately 4.0 dermatologists per 100,000 persons, many rural areas do not meet this standard, reflecting a persistent maldistribution of dermatologic care [5,6]. Limited access to dermatology services may contribute to delayed diagnoses, treatment delays, and disease progression.

Most studies in the United States investigate melanoma incidence and mortality rates at the state or national level, potentially obscuring meaningful variation at smaller geographic scales [7]. Analyses from the Surveillance, Epidemiology, and End Results (SEER) program have demonstrated rural-urban differences in melanoma mortality, stage at presentation, and survival outcomes [8,9]. However, these investigations are typically conducted at national or multi-state levels and do not directly evaluate county-level dermatologist workforce distribution in parallel with melanoma burden within defined regional care landscapes. As a result, state-level analyses may mask important county-level differences, particularly when comparing rural communities. Missouri is a predominantly rural state by land area; a county-level access to dermatologic care study is relevant in this setting [10]. Thus, a more granular, county-level analysis may provide additional insight into regional variation in dermatologist availability and melanoma outcomes across metropolitan and nonmetropolitan communities.

Therefore, this descriptive, county-level analysis evaluated dermatologist availability alongside age-adjusted melanoma incidence and mortality across five selected counties in southwest Missouri using publicly available registry and workforce data, with the goal of better characterizing dermatologic access within a regional care landscape.

## Materials and methods

This descriptive, cross-sectional study examined melanoma incidence, mortality, and dermatologist availability across five counties in southwest Missouri. County-level population estimates for 2018 were obtained from the Missouri Economic Research and Information Center (MERIC) [11]. Age-adjusted melanoma incidence and mortality rates for 2018-2022 were obtained from the State Cancer Profiles database (National Cancer Institute and Centers for Disease Control and Prevention) and standardized to the 2000 U.S. population [12,13].

Rural-urban classification was determined using the 2023 National Center for Health Statistics (NCHS) Urban-Rural Classification Scheme for Counties, which categorizes counties based on Office of Management and Budget metropolitan statistical area definitions and Census population estimates [14]. Under this classification, Greene County is designated as a medium metropolitan county; Jasper and Newton counties are classified as small metropolitan counties; and Barry and Lawrence counties are classified as nonmetropolitan (micropolitan/noncore) counties. Greene County served as the urban reference county in descriptive comparisons.

Dermatologist availability was determined using the National Provider Identifier (NPI) registry [15]. Providers were identified using dermatology specialty taxonomy codes (207N00000X) and assigned to counties based on the primary practice city listed in the NPI registry (Cassville, Springfield, Joplin, Mount Vernon, and Neosho). This approach reflects primary practice location but may not capture cross-county practice patterns. Dermatologist availability was reported as absolute counts and as dermatologists per 100,000 population.

Mortality data were suppressed for counties with three or lower number of deaths in accordance with reporting standards. Confidence intervals for age-adjusted incidence rates are available through State Cancer Profiles; however, given the small number of counties analyzed, the study emphasized point estimates for descriptive comparison.

Analyses were primarily descriptive. An exploratory Spearman rank-order correlation coefficient was calculated to evaluate the association between dermatologist availability and melanoma incidence. Spearman correlation was selected due to the small sample size and potential non-normal distribution of county-level measures. Because only five counties were included, statistical findings were interpreted as exploratory rather than inferential.

County-level measures were summarized in a single table, and choropleth maps were generated to visualize geographic variation. Institutional review board approval was not required because all data were publicly available and deidentified.

## Results

County-level population characteristics, dermatologist availability, and age-adjusted melanoma incidence are summarized in Table 1. Greene County, classified as a medium metropolitan county and designated as the urban reference county, had the highest dermatologist density (9.2 per 100,000 population) and a lower age-adjusted melanoma incidence rate (17.9 per 100,000 population) compared with the other counties in the sample. Greene County was also the only county with reported age-adjusted melanoma mortality (2.5 per 100,000 population); mortality data for the remaining counties were suppressed due to low case counts, precluding meaningful regional mortality comparisons.

**Table 1:** County-Level Population, Dermatologist Availability, and Age-Adjusted Melanoma Incidence and Mortality in Southwest Missouri (2018-2022) Mortality data labeled “suppressed” indicate low case counts in accordance with state reporting guidelines. Data were obtained from the State Cancer Profiles database (National Cancer Institute and Centers for Disease Control and Prevention) and the National Provider Identifier registry.

County	Population	Dermatologists (n)	Dermatologists per 100,000	Age-Adjusted Melanoma Incidence (per 100,000)	Age-Adjusted Melanoma Mortality (per 100,000)
Barry	35,886	0	0.0	20.6	Suppressed
Greene	291,923	27	9.2	17.9	2.5
Jasper	120,636	3	2.5	21.1	Suppressed
Lawrence	38,359	0	0.0	17.8	Suppressed
Newton	58,266	0	0.0	18.2	Suppressed

Barry and Lawrence counties, classified as nonmetropolitan counties, had no identified dermatologists in the National Provider Identifier registry at the time of data extraction. Newton County (small metropolitan) likewise had no providers with a dermatology specialty taxonomy code, while Jasper County (small metropolitan) demonstrated limited availability (2.5 per 100,000 population). Counties reporting zero dermatologists reflect the absence of providers with a dermatology taxonomy code in the registry and do not necessarily indicate the absence of access to dermatologic services in neighboring counties.

**Figure 1:**
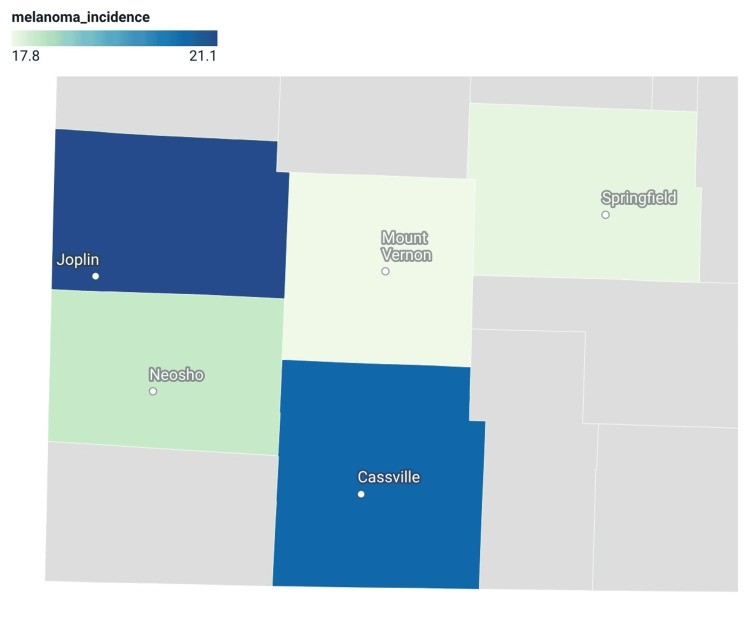
Age-Adjusted Melanoma Incidence per 100,000 Population in Southwest Missouri (2018-2022). Age-adjusted melanoma incidence rates were obtained from the State Cancer Profiles database (National Cancer Institute and Centers for Disease Control and Prevention) and standardized to the 2000 United States standard population. Counties with higher rates are shaded darker.

**Figure 2:**
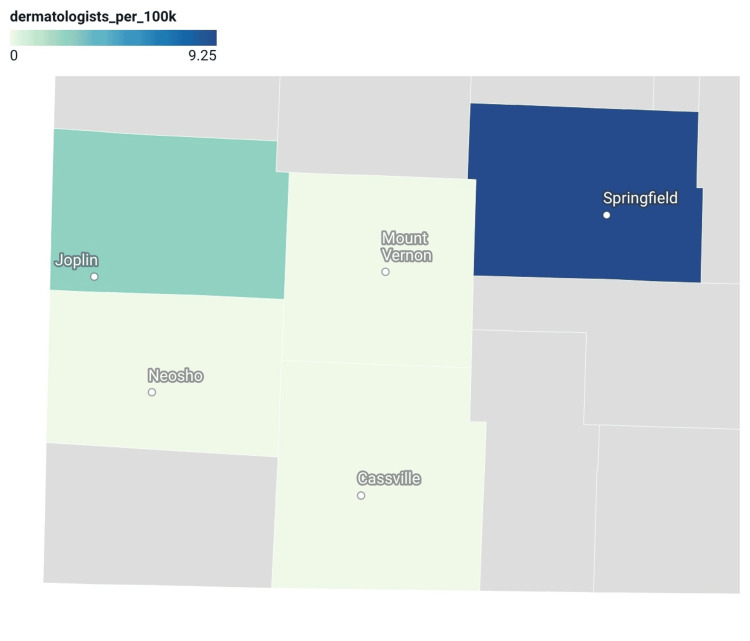
Dermatologist Availability per 100,000 Population in Southwest Missouri. Dermatologist availability was determined using National Provider Identifier registry data and reported per 100,000 population. Providers were assigned to counties based on primary practice city, and values are displayed at the county level, with darker shading indicating greater availability.

The exploratory Spearman rank-order correlation between dermatologist availability and age-adjusted melanoma incidence was weak (ρ=0.11) and is presented descriptively given the small number of counties analyzed. Choropleth maps were generated to visualize geographic variation in dermatologist availability and melanoma incidence (Figures 1, 2). The melanoma incidence map demonstrated higher age-adjusted incidence rates in several nonmetropolitan and small metropolitan counties relative to the urban reference county.

## Discussion

In this descriptive analysis, Jasper and Barry counties demonstrated the highest age-adjusted melanoma incidence rates, while Greene, Lawrence, and Newton counties exhibited similar rates. Because these counties share geographic proximity, they may have similar environmental conditions. Although higher melanoma incidence might be expected to coincide with greater dermatologist availability, this pattern was not observed. Greene County, classified as a medium metropolitan county under the National Center for Health Statistics (NCHS) Urban-Rural Classification Scheme, had the highest dermatologist density and largest population in the study but did not demonstrate the highest melanoma incidence. The weak correlation observed between dermatologist availability and melanoma incidence (ρ=0.11) suggests that provider density alone may not fully reflect melanoma detection patterns. Factors such as occupational sun exposure, socioeconomic disparities, classification heterogeneity across metropolitan and nonmetropolitan counties, and suppressed mortality data may contribute to these findings.

County-level melanoma incidence may also reflect variation in surveillance intensity and diagnostic practices rather than differences in the underlying disease burden. Prior work suggests that rising melanoma incidence may partly reflect enhanced detection practices and case ascertainment rather than changes in risk alone [16]. Increased use of screening, dermoscopy, and lower biopsy thresholds are examples of detection influences that can elevate reported incidence.

Prior work suggests that dermatologist availability alone may not be the primary driver of rising melanoma diagnosis rates. Fleischer Jr. and Aggarwal (2022) proposed that increasing melanoma incidence may reflect enhanced detection of previously unrecognized disease or a true increase in disease occurrence rather than improved access to dermatologic care [16]. In the present regional analysis, several counties classified as nonmetropolitan or small metropolitan demonstrated limited dermatologist availability. Analyses from the SEER program indicate that patients residing in nonmetropolitan counties experience higher melanoma-related mortality and later-stage presentation compared to those in metropolitan areas [8,9]. Regular adherence to dermatologic follow-up after melanoma diagnosis has been associated with reduced melanoma-specific mortality in population-based cohorts, underscoring the importance of facilitating consistent specialty care access [17]. Suppressed mortality data across multiple nonmetropolitan counties in this study further limit the ability to fully characterize regional outcome patterns. Accordingly, mortality differences could not be evaluated within this regional dataset and are referenced here only for contextual comparison with national literature. Collectively, these findings suggest that geographic variation in specialty availability warrants consideration when interpreting melanoma incidence patterns across metropolitan and nonmetropolitan populations.

Differences in dermatologist density may coincide with well-documented barriers to specialty care in nonmetropolitan settings, including longer referral wait times, greater travel distances, and higher out-of-pocket costs [4,6]. National survey data identify these factors as persistent challenges affecting access to specialty services in nonmetropolitan populations [18]. However, Greene County serves as a regional referral hub, and patients may routinely travel across county boundaries to obtain dermatologic or oncologic care. As such, county-level dermatologist density may not fully capture functional access to specialty services. Travel-time analyses, catchment-area modeling, or regional service area approaches may provide a more accurate representation of real-world dermatologic access patterns and should be considered in future investigations.

Several counties classified as nonmetropolitan fell below commonly cited benchmarks for dermatologist density, with some demonstrating no identified dermatologists. Greene County, the regional referral center, was the only county exceeding recommended density thresholds and the only county with reported, non-suppressed melanoma mortality data [5]. Observed gaps in dermatologist availability, coupled with limited mortality reporting, make it challenging to fully characterize melanoma burden in these populations. Variation in dermatologist availability alongside comparable or higher melanoma incidence highlights the complexity of assessing melanoma burden using county-level workforce metrics alone.

This study has several limitations. Given the descriptive cross-sectional design, causal inferences cannot be made and findings should be interpreted as ecological and descriptive rather than reflective of individual-level associations. As an ecological analysis, observed county-level patterns may not represent individual-level relationships, and interpretation should avoid ecological inference. Additionally, the use of publicly available county-level data may obscure important within-county variation. Dermatologist availability was estimated using NPI registry data, which reflects specialty designation and primary practice location but does not measure functional clinical capacity. NPI-based provider counts do not distinguish between full-time and part-time practice, subspecialty focus (e.g., Mohs-only practice), administrative or academic roles, locum tenens coverage, or non-patient-facing responsibilities. As such, provider headcounts may not directly translate to clinical availability. Assignment of providers to counties based on primary practice city may not fully capture multi-site practice patterns or cross-county referral flows, particularly within regional referral networks. Furthermore, access to dermatologic care is influenced by factors beyond provider counts, including new-patient acceptance policies, payer mix, subspecialty distribution, clinic site availability, and appointment wait times, none of which were available in publicly accessible datasets for inclusion in this analysis.

Melanoma mortality data were suppressed for several counties due to low case counts, precluding meaningful mortality comparison across the study region. Additionally, the absence of individual-level clinical data, including stage at diagnosis, treatment timing, referral patterns, and patient outcomes, restricts assessment of disease severity and care pathways. Future studies incorporating unsuppressed mortality data, clinic-level capacity measures, travel-time analyses, and patient-level access indicators may provide a more comprehensive understanding of the relationship between dermatologic access and melanoma outcomes across metropolitan and nonmetropolitan populations.

## Conclusions

This descriptive, county-level analysis examined melanoma incidence and dermatologist availability across five counties in southwest Missouri, with limited county-level mortality reporting. Within this regional sample, the urban reference county demonstrated substantially greater dermatologist availability, yet melanoma incidence was not higher in that setting. Incidence rates were comparable or higher in counties with limited dermatologist presence, while melanoma mortality could not be meaningfully compared due to data suppression in most counties.

These exploratory findings suggest that, in this regional context, dermatologist density alone may not fully represent functional access to dermatologic care or explain observed incidence patterns. However, given the ecological design and limited sample size, these results should be considered hypothesis-generating rather than inferential. Further studies incorporating individual-level clinical data, unsuppressed mortality measures, and more detailed access indicators are needed to better characterize geographic variation in melanoma care and outcomes.
